# Unfolding the layers of LGBTQ+ identity, resilience, and multicultural perspectives in Vietnam

**DOI:** 10.3389/fsoc.2025.1536046

**Published:** 2025-03-18

**Authors:** Huy Lam-Nguyen

**Affiliations:** Adelphi University, Garden City, NY, United States

**Keywords:** gender nonconformity, gender identity, society norms, Vietnamese culture, Asian culture, Western culture, family dynamics, cultural identity

## Abstract

My personal narrative, intertwined with the theoretical acumen of Queer Theory and intersectionality framework, is used within this article as one way of underlining the critical role of lived experiences in forming an insight into LGBTQ+ identity and informing the strategies necessary to support this community. Drawing on Queer Theory, particularly the work of scholars like Jagose, which highlights the fluidity and complexity of identity, this research will analyze the intersectional challenges faced by individuals in specific non-Western regions, particularly in Southeast Asia. These experiences are examined through the employment of a reflexive thematic analysis approach, identifying recurring themes such as (1) gender nonconformity in childhood, (2) the pain of bullying and social pressures, (3) cultural transition and self-acceptance, (4) family support and unspoken understanding, and (5) interaction with LGBTQ+ Communities in Africa and the MENA Region.

## A journey of becoming, 2024

### Part 1: the seed in the sand

In a small town by the sea,

Where the waves whispered softly,

I traded Superman for a Barbie's gown,

Braiding hair, spinning round and round.

Laughter filled the air,

For a moment, the world was kind,

And I was free to fly.

—

**Part 2: storms in the horizon**

Storms on the Horizon

But the wind began to change,

As childhood slipped away,

By grade six, the laughter turned

To words that cut like clay.

*Faggot*, shouted from across the street,

A word that stung,

A shadow on my feet.

It marked the part of me

They did not understand,

Something different, something banned.

I loved a boy with a quiet heart,

In secret, I watched him from afar.

The world said love was only right

Husband and wife, I buried my light.

For years, I held the truth inside,

Until I found the strength to confide.

But when I spoke, he walked away,

And left me with more hurt to stay.

In the halls, they pushed and shoved,

Thinking my body was something to judge.

They held me down to see what was wrong,

Their cruelty silenced my inner song.

I said nothing, just carried the weight,

Unsure how to process their hate.

—

**Part 3: emerging from the chrysalis**

Then came a journey far and wide,

To a place where I could no longer hide.

In a land where freedom sang,

I began to bloom, despite the pain.

Six years of learning to live as me,

Not bound by labels—simply free.

And when my mother's time grew near,

I felt no need to cast away the fear.

Her love had always been enough,

No need for words, no need for labels tough.

She knew me in ways no one could see,

And in that love, I found the key.

Now as I look back, I see

A story of growth, of becoming free.

A child who twirled in Barbie's dress,

Who faced the world's cruelest test.

And through it all, I've come to know,

Identity is like the ebb and flow.

Not something fixed or held too tight,

But a journey through both dark and light.

## Introduction

### Research purpose and objectives

This research is deeply rooted in my personal experiences as an LGBTQ+ individual, examining how these experiences are essential for understanding broader issues of identity, resilience, and cultural dynamics. My journey, from growing up in a small town in Vietnam to cultivating self-acceptance in the United States, provides a distinct perspective on the complexities of LGBTQ+ identity across different cultural settings. The shifts in how my gender expression and sexuality were perceived throughout my life, from early acceptance in childhood to the profound rejection and trauma of adolescence, reflect the challenges faced by many individuals not only locally but also globally. These challenges, closely tied to social norms, cultural expectations, and intersectional identities, are further exacerbated by mental health struggles and the lack of sufficient support systems, particularly in non-Western societies like Vietnam.

The purpose of this research is twofold: *first*, to explore how personal narratives, such as my own, are integral to an understanding of LGBTQ+ issues in specific cultural contexts, including Vietnam and other Southeast Asian countries; and *second*, to develop culturally relevant recommendations for improving mental health interventions and support for individuals across different stages of life. This research moves beyond individual stories by integrating intersectionality and cross-cultural perspectives to offer a more nuanced understanding of how various aspects of identity—gender, sexuality, race, class, and age—shape the experiences of LGBTQ+ individuals. By focusing on non-Western contexts, particularly Southeast Asia, this study addresses gaps in the existing literature on mental health support for people in these regions and proposes interventions tailored to these cultural realities.

### Research questions

This research is driven by three key questions that seek to bridge personal experiences with broader systemic and cultural issues affecting the community.

1. *How are personal narratives of LGBTQ*+ *individuals essential for understanding larger systemic and cultural challenges?*

Personal narratives are not merely connected to but are elemental in understanding how LGBTQ+ individuals navigate their identities within traditional norms, family expectations, and cultural pressures. My own narrative, marked by moments of gender nonconformity, social rejection, and eventual self-acceptance, exemplifies how systemic challenges like heteronormativity, cultural stigma, and bullying impact individuals on a deeply personal level. By analyzing personal stories, this research will uncover how larger cultural and systemic forces shape the lived experiences of LGBTQ+ people. Scholars such as Sedgwick (1990, 2003, 2024) emphasize that individual experiences are inextricably linked to broader cultural narratives and systemic structures, making personal accounts indispensable for analyzing identity formation. The upcoming sections will explore how growing up in Vietnam, being queer, and moving between nations have deepened my understanding of these connections, as well as contradictions, between personal and systemic forces.

2. *How does intersectionality (gender, sexuality, race, age, class) influence the LGBTQ*+ *experience across different regions?*

Intersectionality, as defined by Crenshaw (1991), refers to how various aspects of identity—such as gender, race, sexuality, class, and age—intersect to create unique experiences of both oppression and privilege. For LGBTQ+ individuals, these intersecting identities shape their encounters with discrimination, resilience, and mental health. For example, in Vietnam, cultural expectations surrounding masculinity and traditional gender roles severely punished my gender nonconformity, while in the United States., my gender expression found greater acceptance. In other words, cultural nuances allow me to see myself from different perspectives. This research will examine how intersectional identities shape experiences across different cultural regions, specifically focusing on Western and Southeast Asian contexts. Through this lens, I will probe the relationship between family expectations, social norms, and self-identity, exploring how these dimensions conflict or harmonize based on cultural setting. Readers will see how my journey between Vietnam and the United States illustrates these complexities.

3. *What interventions can be developed to address the mental health needs of LGBTQ*+ *communities across diverse cultures?*

Mental health remains a pressing concern for LGBTQ+ individuals, particularly in non-Western regions where stigma, discrimination, and cultural taboos often hinder access to necessary services. While much research exists on LGBTQ+ mental health in Western contexts, it often overlooks the cultural nuances present in non-Western societies (Meyer, 2003). This research will evaluate the shortcomings of existing mental health interventions and propose culturally sensitive programs tailored to communities in specific non-Western regions, such as Vietnam. It will examine how Western mental health frameworks can fail to account for important factors like class, religion, and family dynamics in non-Western contexts. By incorporating insights from queer methodology and intersectionality theory, this research will provide suggestions for creating mental health interventions that respect cultural differences and effectively address the needs of LGBTQ+ individuals. Differences in class and religion will also be explored to ensure these interventions are as inclusive and adaptable as possible, addressing the diverse realities within communities.

## Literature review

### Cross-cultural perspectives

LGBTQ+ experiences vary significantly within specific cultural contexts, and the nuances of each society shape how identities are perceived and lived. In Vietnam, for instance, traditional values and expectations often present barriers to the acceptance of LGBTQ+ individuals. Research shows that cultural norms in Vietnam frequently marginalize non-heteronormative identities, framing them as outside of conventional family structures and social roles (Mai, 2024; Tyler, 2023). Unlike the broad generalizations about Western societies, where legal reforms and social movements have advanced LGBTQ+ visibility, Vietnam's unique cultural heritage, which emphasizes familial duty and social conformity, creates distinct challenges for people navigating identity and acceptance. Rather than categorizing these experiences as a simple contrast between Western and non-Western, it is important to examine the complexities within Vietnam's cultural framework. Intersectionality, as Crenshaw (1991) notes, is vital in understanding how individuals in Vietnam experience multiple, overlapping identities. These identities—such as being LGBTQ+, Vietnamese, and from a particular socio-economic class—intersect to create unique challenges and forms of resilience. For example, cultural conservatism combined with expectations around familial obligations can intensify the marginalization experienced by these individuals, shaping their paths toward self-acceptance and mental wellbeing (Mai, 2024).

Addressing these challenges requires culturally specific frameworks. The Asian American Queer Studies framework, for example, highlights the importance of placing LGBTQ+ experiences within the broader cultural narratives of Asian communities (Wu et al., 2010). Such frameworks allow for an understanding of how cultural heritage, community values, and intersectional identities influence both identity formation and resilience. By focusing on Vietnam's cultural dynamics and the intersectional nature of identities, this research aims to provide more nuanced insights into the lived experiences of individuals within this context.

### LGBTQ+ identity and resilience

The development of LGBTQ+ identity is a multifaceted process influenced by various cultural and local factors. Cass (1979) introduced a model of sexual identity formation that outlines stages including identity confusion, identity comparison, identity acceptance, and identity pride. Each stage reflects a progression in self-awareness and acceptance of oneself, highlighting the impact of external social pressures. Recent studies have built on Cass's framework, suggesting that identity formation is not linear but rather a dynamic and ongoing process (Schwartz et al., 2013).

Queer Theory serves as a crucial lens for understanding LGBTQ+ identities, challenging traditional binaries, and allowing for the exploration of diverse expressions of gender and sexuality. Jagose (1996) asserts that Queer Theory emphasizes the fluidity of identity, which is particularly relevant in contexts where rigid definitions can be harmful. This perspective encourages the examination of personal narratives within broader constructs, providing insight into how LGBTQ+ individuals navigate their identities amidst cultural expectations (Sholock, 2007). Additionally, the work of Crenshaw (1991, 2013) on intersectionality highlights the necessity of considering how overlapping identities—such as race, gender, and class—affect the experiences of LGBTQ+ individuals, particularly in varied cultural contexts (McCall, 2005).

### Mental health in LGBTQ+ communities

LGBTQ+ mental health services in Western countries, particularly in the United States and parts of Europe, are generally more developed and accessible. Over the past few decades, there has been a growing recognition of the specific mental health needs of LGBTQ+ individuals in the West, leading to the creation of specialized clinics, support groups, and mental health programs (Meyer, 2015). These resources are often designed to address the unique challenges faced by LGBTQ+ individuals, including public discrimination, family rejection, and minority stress. The United States., for instance, has established numerous mental health programs aimed at supporting youth and adults, providing services such as therapy, support groups, and crisis intervention specifically tailored to the needs of this population (Russell and Fish, 2016). Additionally, legal protections in Western countries, such as the legalization of same-sex marriage and anti-discrimination laws, have contributed to an environment where LGBTQ+ individuals are more likely to feel supported and validated, which has a positive impact on mental health outcomes (Herek, 2006, 2009).

However, while Western countries offer more comprehensive mental health services for LGBTQ+ individuals, there are challenges related to the Westernization of mental health research and practices. The prevailing models of mental health in the West often emphasize individualism, autonomy, and self-expression, which may not align with the cultural values of non-Western societies such as Vietnam, where collectivism and familial obligations are more central (Russell and Fish, 2016). This can lead to a mismatch between the mental health needs of communities in non-Western contexts and the frameworks through which mental health services are offered. For instance, therapeutic approaches that prioritize coming out as a key to mental health and wellbeing may not resonate with individuals in collectivist cultures, where coming out could jeopardize family relationships and social harmony (Nguyen and Angelique, 2017). This highlights the need for culturally sensitive mental health services that take into account the specific social and familial dynamics of different cultural contexts.

Moreover, the *WEIRD* bias in psychological research significantly impacts the understanding of mental health within LGBTQ+ populations. This term—referring to Western, Educated, Industrialized, Rich, and Democratic populations—underscores the limitations of research that predominantly focuses on these groups, often neglecting the diverse experiences of LGBTQ+ individuals in non-Western societies (Henrich et al., 2010a). Studies have shown that mental health research often overlooks the unique cultural contexts that shape the lived experiences of LGBTQ+ individuals, leading to an incomplete understanding of their mental health needs (Budge et al., 2013). Existing gaps in mental health support are evident, particularly in addressing issues beyond AIDS-related concerns. While historical research has primarily focused on the psychological impacts of the AIDS epidemic, contemporary literature underscores the need for broader mental health services that address anxiety, depression, and trauma within communities (Meyer, 2003; Timmins et al., 2017). This is particularly pressing in non-Western contexts, where stigma and discrimination can exacerbate mental health challenges (Mai, 2024). Specifically, conversations about mental health in a Vietnamese context are completely rare, if not absent.

As a result, there is a growing recognition of the importance of culturally competent mental health interventions tailored to LGBTQ+ individuals. For instance, the incorporation of community-based approaches can enhance mental health services, ensuring they are sensitive to cultural norms and values (Meyer, 2003). Such interventions are crucial for effectively addressing the mental health needs of diverse populations.

### Challenges in Vietnam

In Vietnam, the mental health needs of LGBTQ+ individuals are frequently overlooked due to several interrelated factors, including a lack of resources, social stigmas, and inadequate healthcare infrastructure. While Vietnam has made strides in LGBTQ+ rights—such as lifting the ban on same-sex marriage in 2015, although without legal recognition of same-sex unions—there remains a significant gap in mental health services that cater specifically to the LGBTQ+ population (Phuong, 2022). Mental health services in Vietnam are still largely underdeveloped, often treated with a mixture of traditional and biomedical practices, and specialized services or trained professionals are scarce (Dang et al., 2020). For LGBTQ+ individuals, this situation is exacerbated by the fact that their sexual orientation and gender identity are still seen as taboo topics within Vietnamese society.

The stigmatization of mental health issues is widespread in Vietnam, with many individuals reluctant to seek professional help due to fear of being perceived as weak or mentally unstable (Do et al., 2014). This stigma intersects with the marginalization of LGBTQ+ identities, adding additional layers of discrimination. The pressure to conform to traditional gender roles and heteronormative expectations can lead to significant mental health struggles, including anxiety, depression, and internalized homophobia (Russell and Fish, 2016). Many individuals in Vietnam feel compelled to hide their identities, which exacerbates feelings of isolation and distress. The cultural expectation of *filial piety*—where children are expected to uphold the family's honor and wellbeing—creates immense pressure on LGBTQ+ individuals, many of whom feel that their nonconforming identities disgrace their families (Jacka, 2010; Nguyen and Angelique, 2017). This pressure can lead to increased mental health issues, as individuals may grapple with feelings of guilt and fear of rejection, further compounded by a lack of supportive mental health services.

### Healing-centered engagement in mental health research

One of the key issues in the field of LGBTQ+ mental health research is the dominance of studies that focus on populations that are Western, Educated, Industrialized, Rich, and Democratic (WEIRD). This term, coined by Henrich et al. (2010b), describes the tendency for psychological research to disproportionately rely on samples from these WEIRD societies, leading to findings that may not be generalizable to non-Western populations. In mental health research, this WEIRD bias manifests in several ways. Much of the research is conducted in Western countries, particularly in North America and Western Europe, where the social, political, and cultural conditions differ significantly from those in non-Western contexts (Meyer, 2015). As a result, the mental health challenges faced by LGBTQ+ individuals in countries like Vietnam, where acceptance is lower and resources are more limited, are often underexplored or misunderstood. For instance, studies on minority stress—a prominent framework used to explain the mental health disparities faced by minority groups—are largely based on Western populations (Meyer, 2003). While minority stress theory has provided valuable insights into the impact of discrimination and stigma on mental health, its focus on individual experiences of stress may not fully capture the collective and family-oriented experiences of LGBTQ+ individuals in non-Western societies. In these contexts, the sources of stress may be more closely tied to family and community dynamics rather than solely individual experiences (Russell and Fish, 2016). Moreover, the WEIRD bias raises concerns about the applicability of Western mental health interventions in non-Western contexts. Many therapeutic approaches and mental health interventions developed in the West, such as cognitive-behavioral therapy (CBT) and other individualized forms of therapy, may not resonate with LGBTQ+ individuals in cultures where mental health is viewed through a more collective or holistic lens (Van Vu et al., 2022). For example, Vietnamese people may prefer and benefit from community-based or family-centered interventions that involve the support of their social networks rather than focusing solely on individual therapy sessions (Nguyen and Angelique, 2017).

To address this WEIRD bias, researchers must prioritize conducting studies that include diverse, non-Western populations and develop culturally sensitive frameworks for understanding LGBTQ+ mental health. This includes recognizing the unique challenges faced by LGBTQ+ individuals in different cultural contexts and tailoring interventions to meet their specific needs. Without this, the global understanding of LGBTQ+ mental health will remain incomplete, and many individuals in non-Western contexts will continue to be underserved. A promising approach to bridge this gap is the model of *Healing Centered Engagement* proposed by Ginwright (2018). This framework emphasizes the importance of community, culture, and collective healing, rather than solely focusing on individual pathology. *Healing Centered Engagement* seeks to empower individuals and communities by acknowledging their strengths and resilience while addressing the historical and systemic factors that contribute to their struggles. In the context of LGBTQ+ mental health in non-Western societies, incorporating *Healing Centered Engagement* could provide a pathway for developing interventions that align with cultural values and community dynamics. By centering healing within the community context and emphasizing the interconnectedness of individuals, this model could foster a more holistic understanding of mental health that resonates with communities facing unique challenges.

### Queer methodology and intervention strategies

Queer theory, as articulated by scholars like Jagose (1996), offers a critical framework for understanding the diverse identities and experiences of LGBTQ+ individuals, particularly in non-Western contexts like Vietnam. It challenges normative assumptions about gender and sexuality, emphasizing fluidity and multiplicity in identities. This perspective is vital for examining how traditional gender norms are deeply rooted and highly defined in Vietnamese society, where sexual diversity remains stigmatized. People with diverse sexual orientations and gender identities in Vietnam often navigate social expectations that compel them to conform to rigid gender norms, leading to marginalization not only based on sexuality but also due to intersecting factors such as class, race, and cultural background (Jagose, 1996). For these individuals, gender nonconformity can result in social ostracism, compounded by challenges such as family rejection, lack of legal protections, and limited access to gender-affirmative mental health services. The application of queer theory sheds light on how these pressures intensify mental health risks when they may feel compelled to hide their identities in a culture that prioritizes family honor and social cohesion.

In this context, culturally sensitive intervention strategies are essential for promoting mental health among LGBTQ+ individuals. A promising approach is the development of community-based mental health interventions. These initiatives engage local communities to create safe spaces where LGBTQ+ individuals can receive support without fear of discrimination. Incorporating family members and local leaders into these programs can foster acceptance and reduce stigma at the community level. Educational campaigns that emphasize the importance of mental health and human rights within a Vietnamese cultural framework can be particularly effective in breaking down barriers to acceptance. Digital health solutions also offer a significant opportunity to bypass local and logistical barriers to accessing mental health services.

The concept of positionality, which recognizes how one's social and cultural identity shapes research perspectives and outcomes, is crucial in this discourse (England, 1994). As a non-binary person from Vietnam, my identity informs my work by grounding it in lived experience. This positionality allows me to navigate social issues with an insider's understanding of the cultural constraints in Vietnam while employing queer theory to challenge normative frameworks. My experiences navigating gender expectations and social rejection also shape my perspective on the importance of culturally sensitive mental health interventions. While Western mental health interventions often emphasize coming out as crucial for psychological wellbeing, in Vietnam, the focus may need to shift toward managing internal conflict in ways that preserve family relationships. By advocating for contextually appropriate interventions that balance individual identity exploration with family and community dynamics, we can create a hybrid model of intervention that is culturally relevant and scalable. Furthermore, combining technology with community-based approaches, as seen in the hybrid model of intervention, provides a pathway for mental health support for LGBTQ+ individuals across diverse contexts, ensuring that interventions address the unique challenges faced by these populations while promoting overall mental wellbeing.

## Analysis of *A Journey of Becoming*

### Theme 1: gender nonconformity in childhood

My early childhood was composed of one self-expression after another, those moments later forming sources of both joy and pain. I remember the freedom I felt playing with Barbies and trying on wigs, enjoying the novelty of changing into characters that somehow felt truer to my inner sense of self. These would be the toys that generally catered to girls, where I could explore a fluid and comfortable gender identity long before I had the language to define it. My early times were marked by a sense of difference that lingered beneath the surface, which reflects the non-linear journey of queer identities. Stockton (2009) describes this as *sideways development*, where understanding of self emerges through detours rather than a straightforward, socially ingrained path. Stockton's framework on the queer child's sense of feeling something different also offers a lens into how my early experiences of queerness were shaped by creative exploration rather than explicit articulation. My fascination with fashion and with the aesthetics of femininity was surprisingly met with encouragement from my family—a quiet yet significant acceptance that felt unique considering the conservative environment in which I was being raised. It is in the family unit that most Vietnamese find their identity, with the parents taking a very protective role in the children's behaviors and expressions. The subtleness of my family's acceptance was not only an outlier but, more importantly, it spoke to traditional Vietnamese values' complexities that sometimes can accommodate nuance.

Not until I was in elementary school did I feel my interests were somehow not *normal*; as my friends considered these hobbies a departure from the gender roles that society, particularly schools, forced and molded students into. Vietnamese education, like most other heteronormative systems in the world, does enforce very strong notions about early-age gender conformance. By middle school, this had turned into active ridicule. My actions and non-conformity became targets for bullying and exclusion. Research by Kane (2006) further illustrates how gendered expectations start to take shape early in childhood and any deviations are usually socially spurned. In my case, traditional values that centered on familial duty and conformity, protective as they were within the family environment, became stifling within the school environment as the rigid gender binary was instituted, and I was forcibly made cognizant of a difference.

This contrast between the acceptance I received at home and the ostracism I met with at school reflects broader cultural and historical tensions within Vietnam. While family may be a source of refuge in many people's lives, more often than not, public attitudes, which have been informed by Confucian understandings of order and gender, have tended to marginalize and stigmatize those who have transgressed such culturally ascribed identities. This framework, with a focus on filial piety, respect for authority, and conformity to a strict hierarchy, often dictates the definition of appropriate behaviors. The deeply ingrained dichotomy is important to understand in grasping how the LGBTQ+ community navigates identity in Vietnam, which reinforces the values of family duties and social pressures. Furthermore, Vietnamese political history, particularly during and after colonization and the Vietnam War, has shaped public attitudes toward conformity and dissent. The emphasis on collectivism and national unity, alongside the legacy of a one-party system, has created an environment where deviation from established norms is not only frowned upon but can also lead to social ostracization.

As I grew older, my understanding of identity was now heavily shaped by traditional gender roles, with clear expectations for how individuals should express their gender and sexuality. Masculinity is often equated with strength, control, and dominance, while femininity is associated with submission and care (Luong, 2003). Vietnam's collectivist culture also emphasizes familial duty and community harmony, meaning deviations from the norm are often viewed as threats to family honor and social cohesion (Nguyen and Angelique, 2017). This context made my early experiences with gender expression and sexual orientation particularly fraught. I internalized a deep sense of shame about my nonconformity, feeling as though my authentic self was somehow an affront to the values I had been raised to uphold. This tension is reflective of the findings of researchers such as Manalastas et al. (2017), who has explored how LGBTQ+ individuals in Southeast Asian societies often face a double burden of conforming to both familial and societal expectations. Fortunately, my family is an exception and my interaction with them is not typical of the overall experience of others in the community.

### Theme 2: violence and social pressures

Middle school inaugurated a distressing time of bullying, where my *failure* to conform to the standards of traditional gender roles became an easy focal point for perpetrators. I was often referred to as *freak* or *pervert* (translated to as *biến thái* in Vietnamese) and *sissy* (translated to as *bóng lộ* in Vietnamese)—terms that used the vilest forms of homophobic innuendos. The bullying I suffered was not confined to mere name-calling but went as far as hurting me emotionally and sometimes even physically. It goes in tune with such studies as Kosciw et al. (2020) that focus on the disproportionate rates of bullying for LGBTQ+ youth within educational institutions. It was not just my peers, from teachers to other personnel of authority, implicit expectations were always there to modify my behavior to forcibly make me heteronormative. What made this experience particularly traumatic was when my body turned into an experiment for my classmates. They did not even tease me across the room but dissected my body from some cruel curiosity as if there was something wrong with me and they needed to find it. It had crossed over the threshold of insults to invasive touching under the guise of *figuring out* what was wrong with me. They touched me, probed me, and violated my boundaries to render my body a reason for their jests and an object of experimentation as if their hands were trying to solve the puzzle of my existence. It was not mere bullying but a horrific way of sexual curiosity aimed at dehumanizing me.

These physical violations were not anything to do with curiosity or misunderstanding but a performance of power that meant to demean and strip me of dignity and identity. My body was rendered a site for their investigation as if they sought to find and confirm the *wrongness* in them being pointed out by me. It was not an isolated incident but a string of violations that merely added to the emotional hurt caused, making me feel like an object that had been shattered into pieces. This inappropriate touching and *experimentation* exemplifies Foucault's (2003) concept of biopower, where social hierarchies and norms enforce control over individuals. These acts go beyond mere coercion and indicate a larger cultural dynamic in which queer bodies render sites of power struggles and imposition of heteronormative dominance. Additionally, the personal violations I faced were deeply tied to Foucault's (1977) and Foucault et al.'s (1978) concept of surveillance, where the gaze of peers and the pressure to conform to social norms functioned as tools of control. The constant monitoring and invasive actions underscored how queer bodies are policed in daily interactions. These acts were emblematic of a broader culture that seeks to regulate and discipline those who deviate from prescribed gender and sexual norms, perpetuating cycles of trauma and exclusion.

It was during this time that I also dealt with unrequited love—a relatively complicated part of my identity. I developed feelings for a male classmate, but the social pressure of concealment meant these emotions had to be shrouded in secrecy. When I finally did come out with them, the response to deal with was an awkward rejection that became the fodder for gossip amongst my cohorts. What was private was now public, the amplification of my vulnerability, coupled with feelings of shame and isolation, which were already intense. This reaction merely deepened my sense of alienation and resulted in the repression of my desires. This unreturned love added to the emotional burden furthered by the rejection of society, pushing me further into a deeper sense of self-hatred and thus mirroring the symptoms of minority stress that Meyer's (2003) theory described among LGBTQ+ individuals. In other words, there exists a chronic psychological burden furthered by social stigma.

This traumatic incident I experienced in high school was part of years of bullying and violence. This was physical abuse by some of my classmates, which deprived me of my bodily autonomy and cast a deep scar on my consciousness. The scene was one of extreme humiliation—the already vulnerable child was taken advantage of by peers to reinforce their otherness even more. The moment was deep in assaulting my sense of self and integrity of the body, representing a fractured relationship with the body. Literature on trauma and body autonomy, particularly among LGBTQ+ individuals, shows how such violations often have significant impacts on self-perception and identity, leading to long-term psychological harm (Grossman and D'Augelli, 2007; Herman, 1992; Stocks, 2007). What happened to me marked a turning point in my journey-a moment in time that devastated me but eventually fueled my resilience to begin what was sure to be an arduous process of healing.

### Theme 3: cultural transition and self-acceptance

Coming to the United States to study in Brooklyn, New York was a profound turn in the journey of self-discovery. In contrast with the conservative, heteronormative atmosphere of my hometown, Brooklyn started to become the space that allowed more acceptance, where I could finally start exploring my identity openly. The cultural environment of this vibrant borough allowed me to encounter people who embraced diverse expressions of gender and sexuality, which seemed unprecedented to me. This atmosphere empowered me to shed layers of internalized shame and self-repression that had suffocated me for years. For the first time, I finally felt my identity was something to be celebrated, not erased. While my experience in Brooklyn and surrounding areas highlighted the transformative power of affirming spaces, it also underscored the disparities within the host country. It is imperative to recognize that not all states offer similar opportunities for acceptance. Areas such as Florida or Tennessee, for instance, have seen ongoing backlashes against the community, particularly targeting young transgender individuals (American Civil Liberties Union, 2024; Human Rights Campaign, 2024).

This geographical context aligns with what Braidotti (2011) and Haraway (1991) discuss through the term *politics of location*, which is acknowledging how physical spaces shape the identity (re)formation process. Brooklyn, with its progressive stance on LGBTQ+ issues, became a venue for my unlearning/unbecoming social constraints imposed in my home country. Such a contrast between my experiences in Vietnam and the United States reveals how identity formation does not happen in isolation but is deeply embedded within cultural, political, and economic forces. This is also consistent with Butler's (1988, 2002) concept of performativity and D'Augelli's (1994) life span model of lesbian, gay, and bisexual identity development, which emphasizes how the social environment plays a crucial role in identity formation.

Besides, migration, for me, was not merely a geographic relocation but a bodily reorientation, as indicated in Gorman-Murray's (2007) *Rethinking Queer Migration through the Body*. The interplay between space and queer identity further resonates with Valentine and Skelton's (2003) work on paradoxical queer spaces. While the anonymity of urban landscapes in Brooklyn allowed me to explore facets of my identity, the hypervisibility of certain queer spaces sometimes perpetuated new forms of exclusion based on race and class. Many times, I felt I was at an intersection between my ethnic and sexual identities, trying to make sense of the values of my culture against progressive views on gender and sexuality here in the host country. One stark example of this was when working with my peers on the identity discussions, there was an assumption that my experiences and beliefs were the same, if not identical, to other people in the United States who identified as LGBTQ+. This genuinely minimized my background altogether. Besides, in one Pride Month discussion, I was surrounded by utterances of joy and celebration but found the struggle of articulation burdensome to describe how pride was different in my experience, as it was shaped by my cultural background and the residues of traditional expectations. Another example was when I attended a *Diversity and Inclusion* event in my community. It was highly vibrant and welcoming, yet there was some kind of dissonance in me, as in discussions on sexual liberation that sometimes sounded alien from where I came from. It was the fetishization of non-Western identities and putting people out as objects based on certain characteristics that made me understand how my identity could be both marginalized and reconstituted according to others. In other words, with LGBTQ+ spaces in the United States often dominated by white, middle-class individuals, I sometimes felt marginalized due to my Asian heritage and the specific cultural experiences I carried with me from Vietnam. This sense of alienation is reflective of the broader issue of racial exclusion within the community, where people of color often feel that their identities are either erased or tokenized (Battle and Ashley, 2008; Narvaez et al., 2009).

Furthermore, the intersection of class and identity has played a significant role in shaping my experiences. Growing up in a working-class family in Vietnam, financial concerns were always at the forefront of my family's life, and my ability to express my identity was often constrained by economic limitations. Even in the United States, socioeconomic status continues to impact the resources available to me as a non-binary person, particularly in terms of access to healthcare and mental health services that affirm my gender identity. For example, the high cost of therapy and lack of inclusive healthcare options created barriers to receiving the support I needed to navigate my experience. This reality emphasizes the urgent need to address both socioeconomic disparities and the unique challenges faced by individuals in our community in accessing affirming care and resources. Indeed, studies show that LGBTQ+ individuals from lower-income backgrounds often face additional barriers in accessing necessary care, further compounding the challenges of navigating their identities (Burwick et al., 2014).

### Theme 4: family support and unspoken understanding

While many LGBTQ+ individuals face familial rejection, I consider myself privileged to have the quiet support of my family. My mother's illness in 2022 marked a particularly poignant moment in our relationship. Although I never felt the need to *officially* come out to her, there was an unspoken understanding between us regarding my identity. This mutual recognition reflects research suggesting that, in certain cultures, explicit conversations about sexuality may be unnecessary for acceptance (Yip, 2005). In Vietnam, where open discussions about identities are often rare or even absent, my family's quiet support becomes even more significant. The cultural context tends to emphasize privacy, leading to a preference for keeping personal matters, especially those related to sexuality, within the family. This tendency often results in a delicate balance between public and private identities, where acknowledging one's LGBTQ+ status can feel risky. For many, coming out represents a radical departure from deeply ingrained cultural norms that prioritize family harmony and social conformity. The experience of keeping private thoughts concealed can create a significant sense of isolation, especially when social values reject the legitimacy of non-heteronormative identities. My journey illustrates how *coming out* can carry different meanings within this cultural framework. In my case, it was less about a formal declaration and more about a quiet acknowledgment of my identity that my family and I understood without needing to articulate it explicitly.

When my mother fell ill, our relationship deepened in ways that transcended traditional expectations. The circumstances of her health brought us closer, revealing that her love for me surpassed any social pressures to conform to traditional values. This unspoken acceptance serves as an unearned privilege that many LGBTQ+ individuals, particularly in conservative cultures, may not experience (Ching et al., 2018). While some LGBTQ+ individuals may grapple with the denial of their identities due to external pressures, I found solace in knowing that I was loved and accepted within my family, even if our discussions remained implicit. This quiet acceptance allowed me to embrace my identity without the burden of formal acknowledgment, a privilege that starkly contrasts with the more public battles for acceptance that many face in less supportive environments. In this way, my experience highlights the complexities of navigating private vs. public identities within a cultural landscape that often favors silence over open dialogue about LGBTQ+ issues.

### Theme 5: interaction with LGBTQ+ communities in Africa and the MENA region

During my time working with a non-profit organization, I had the opportunity to engage with LGBTQ+ refugees and asylum seekers from Africa and the MENA (Middle East and North Africa) region. Their stories were marked by unimaginable struggles, as many of them faced extreme danger and persecution in their home countries simply for expressing their sexual or gender identity. These individuals had to flee due to war, political instability, and violent homophobia, a reality that is well-documented in the literature on LGBTQ+ refugees (Shidlo and Ahola, 2013). In these regions, coming out or being outed often carries life-threatening consequences, far beyond the social stigma faced in more liberal countries.

Many of the people I worked with had been disowned by their families, exiled from their communities, and were constantly living in fear. Safe Place International provided them with a sanctuary where they could begin to rebuild their lives. The theme of belonging, so central to LGBTQ+ identity, was complicated for these individuals. While they had found temporary refuge, they were also displaced, cut off from the homes and communities that had once been a part of their lives. Their resilience was awe-inspiring, as they navigated a reality where safety was constantly at risk.

These experiences illuminated the stark differences in the nature of LGBTQ+ struggles between the Global South and the West. While LGBTQ+ communities in Western countries often contend with discrimination and mental health challenges, individuals in regions like the MENA (Middle East and North Africa) and parts of Africa face existential threats that can be life-threatening. In many of these areas, same-sex relationships are criminalized, leading to severe legal repercussions, violence, and even death. The stakes are much higher; survival often becomes the primary concern, as people must navigate not only social rejection but also legal systems that actively seek to punish them.

I recall conversations with LGBTQ+ asylum seekers from these regions, which opened my eyes to their profound struggles. One conversation that stands out was with a young man from Egypt who shared his harrowing experience of being outed by a friend. He explained how a simple act of intimacy—holding hands with another man—was seen as a crime, leading to violent reprisals not just from the authorities but also from his community. He recounted how, after being discovered, he had to flee to escape legal punishment as well as the very real threat of mob violence. This conversation underscored the terrifying reality that for many, sexual activity can lead to brutal consequences, further complicating their quest for love and acceptance. Similarly, I spoke with a woman from Uganda who described how her identity put her in constant danger. She recounted a time when she and her partner were ambushed by a group of men who had been monitoring their activities. In her words, *In Uganda, love can be a death sentence*. Her story made me reflect on the potential for similar violence in Vietnam, where social norms are also heavily influenced by traditional values. Although Vietnam does not criminalize same-sex relationships, there remains a pervasive culture of silence and stigma that could lead to violent reactions if individuals are discovered engaging in non-heteronormative behaviors.

Literature on LGBTQ+ asylum seekers emphasizes their vulnerability, particularly when navigating asylum processes that often overlook the intersection of sexual identity and geopolitical context (Millbank, 2009). Many of these individuals are forced to navigate a complex web of legal requirements while simultaneously dealing with the trauma of their past. Despite these extreme difficulties, they often find strength and solidarity within their communities. Through mutual support, they carve out spaces of belonging, creating networks of resilience even in the most hostile environments. This sense of community becomes crucial, providing not just emotional support but also practical assistance, as individuals learn to advocate for themselves and one another in the face of overwhelming odds.

## Recommendations

### Mental health support across cultures and demographics

Any improvement in the mental health outcomes of LGBTQ+ people therefore needs the inclusion of strategies that take into account unique challenges arising from diverse age groups, in addition to intersecting factors related to class and religion. Differences in the degree of acceptance or amount of resources may lead to variations in mental health and wellbeing for each demographic.

Regarding age dynamics, interventions for *LGBTQ*+ *youth* should be based on education, peer support, and safe spaces promoting the exploration of identity in a safe environment without fear of social rejection. School-based programs will become essential to this population, including services for mental health, policies against bullying, and counseling that is affirmative to LGBTQ+. Empowering in a way, these programs could enable young people to better cope with their identities and help build resilience against adversities (Russell and Fish, 2016). Moreover, the sensitization of teachers, parents, and other staff would provide a very conducive environment both at home and in schools, generally improving the social climate in which LGBTQ+ youth are living. For *middle-aged LGBTQ*+ *adults*, however, relationship dynamics, social acceptance, workplace, and family settings are the points of concern for them. Workplace-based mental health support and strong anti-discrimination policies are features that form the basis of settings allowing people to be themselves without persecution. According to Meyer (2015), the facilitation of workshops on workplace diversity and sensitivity training could foster an atmosphere of tolerance and respect where the mental health burden from concealing one's identity would be reduced. Besides, mental health interventions for *LGBTQ*+ *older adults* should pay particular attention to the heightened consequences that a lifetime of discrimination and marginalization can have. This requires special care that addresses both physical and mental health needs for this population, which is at risk for certain aging and social isolation-specific issues (Fredriksen-Goldsen et al., 2013). Programs providing social engagement and a sense of community, such as support groups and recreational activities, have the potential to greatly improve the quality of life for older adults in the community.

One cannot but note the role of class in shaping mental health outcomes—individuals in the lower socioeconomic classes, in addition to barriers to acceptance, have lesser access to most mental health resources and increased levels of stressors emanate from such financial insecurity. Interventions should ensure that affordability and accessibility come first so that all members of the community enjoy mental health support services, regardless of socioeconomic status. Furthermore, religion also features prominently in the creation of LGBTQ+ experiences, where faith communities can be sources of both acceptance and rejection. Interventions for individuals from religiously endemic backgrounds should be sensitive to the interaction of faith with sexual identity. Resources that promote the conduct of discussions within faith communities on the acceptance of LGBTQ+ individuals will go a long way in helping to diminish stigma and build acceptance. Workshops and discussions also include religious leaders and advocates to allow for reconciliation and support in keeping spiritual beliefs without renouncing one's sexual identity.

### Cross-cultural approaches to advocacy

Cross-cultural advocacy must strike a very delicate balance between respect for local culture and the demands to push for greater inclusivity and equality. A powerful framework in doing this is through partnerships with local activists and organizations to ensure deeply culturally situated advocacy. In a country like Vietnam, where the roots of social life are based on family and community, the strategies for advocacy may involve educating the families about LGBTQ+ identities and fostering acceptance within these units. This way, not only is the respect paid to the cultural norms but also encourages gradual change from within the community.

Historically, solidarity in the Vietnamese context has been substantially modified in meaning and practice under the strong influence of the sociopolitical landscape and the impact brought about by various historical events. During the Vietnam War, solidarity was largely framed in terms of the struggle against colonialism and the fight for national unity. In this context, as Vietnam took on more explicit characteristics of a socialist state, notions of solidarity were made along the lines of class struggle and collective identity, often to the detriment of concern for issues of sexual orientation and gender identity. Thus, rights concerning LGBTQ+ people were highly marginalized, trumped by general political concerns. However, over the last couple of decades, the notion of solidarity among LGBTQ+ individuals in Vietnam has changed. Notably with more social movements, the idea of solidarities not only around class, religion, and ethnicity but also around gender and sexuality has been increasingly taken into consideration by a new generation of activists. It is manifested in the emergence of more grass-rooted organizations and community-based initiatives centering around the cause of advocacy for LGBTQ+ rights, with increased demands for space that allows an individual to be acknowledged and celebrated for who they are.

International organizations, like Safe Place International, where I have had the opportunity to work with, provide important lessons about how to best support LGBTQ+ individuals in highly conservative societies. Where coming out often means putting oneself at a high risk of violence or displacement due to the political or religious pressure in the region, advocacy has to make safety and confidentiality a priority. These underground networks and community-based organizations provide safety and a sense of belonging that is very important to most LGBTQ+ individuals. All this can be extended to create global networks that can facilitate more support and advocacy to help empower LGBTQ+ people with the kind of wherewithal they need to negotiate oppressive ecologies while simultaneously agitating for broader systemic change.

## Conclusion

Reflecting on my lived experiences, I have used my personal stories to explore the intersections of identity, culture, and resilience. My journey, from growing up in a conservative environment in Vietnam to seeking a sense of belonging in affirming spaces like Brooklyn, has served as a platform to highlight the profound differences in LGBTQ+ experiences across cultural contexts. These stories have not only allowed me to make sense of my own identity but also to connect individual experiences to broader, more complex discourses surrounding LGBTQ+ mental health and wellbeing. I hope to provide an intercultural lens through which we can understand how identity formation is shaped by the cultural and social environments individuals navigate on their own terms. In this way, my experiences become a means to offer insight into interventionist practices necessary to foster safer and more accepting spaces for LGBTQ+ individuals, especially those from marginalized groups.

Identifying as part of the LGBTQ+ community cannot be understood in isolation from the cultural context in which people are deeply embedded. In the case of Vietnam, due to traditional and cultural stigmatization associated with mental health and identity, culturally sensitive interventions become urgent in fostering acceptance and improving mental health outcomes. In this context, Western models of LGBTQ+ identity and access to mental health services do offer some useful lessons, but their generalizability depends on the ability to accommodate these cultural contexts within a non-Western framework. Research that uses an intersectional framework for examining the axis of identity markers such as gender, sexuality, race, class, and culture brings into discussion a far more nuanced understanding of struggles and privileges experienced by LGBTQ+ individuals across the world. For instance, the community in Vietnam has to negotiate intersectional difficulties within cultural, family, and institutional pressurization that are amenable to tailored community-based interventions. In turn, Western contexts may boast more institutionalized support systems but fail in their ways to overcome intersectionality, especially related to marginalized subgroups within the LGBTQ+ community. Such gaps can only be bridged by cross-cultural research and collaboration toward more inclusive holistic mental health frameworks.

The future of mental health for the community requires inclusive, culturally sensitive, and accessible interventions. Digital health solutions signal a new direction in reaching the most marginalized LGBTQ+ communities, where the service provision for essential mental health support may be scant or stigmatized. These digital platforms assure anonymity, confidentiality, and a sense of community. These could also be scaled-up models by infusing technology into community-based interventions and modified to conform to the cultural ecology in which they would exist. Lastly, future mental health interventions will need to consider diversity within the broad coalition of LGBTQ+ individuals by addressing the unique needs of different age groups, as well as racial and ethnic minorities living both in urban and rural settings. Mental health support needs to be revised in consideration of an intersectional approach, recognizing the fluid and multifarious ways oppression and marginalization occur that shape the mental health experiences of LGBTQ+ people. I am optimistic that as we come together for a commitment to culturally responsive care and advocacy, we can create a world where LGBTQ+ people everywhere can have access to needed support to thrive.

## Data Availability

The original contributions presented in the study are included in the article/supplementary material, further inquiries can be directed to the corresponding author.
